# Guipi Decoction Attenuates Fibrosis and Oxidative Stress to Ameliorate Diabetic Erectile Dysfunction in Rats *via* the RhoA/ROCK Signaling Pathway

**DOI:** 10.2174/0118715303420396251202100011

**Published:** 2026-03-17

**Authors:** Longhao Sun, Jiayun Li, Yuexin Ma, Xu Wang, Zhuo Zhao, Zhanmin Li

**Affiliations:** 1 Shenyang Jinghua Hospital Co., Ltd., Shenyang, Liaoning 110001, China;; 2 The Second Clinical College, Liaoning University of Traditional Chinese Medicine, Shenyang, Liaoning 110847, China;; 3 Affiliated Hospital of Liaoning University of Traditional Chinese Medicine, Shenyang, Liaoning 110032, China

**Keywords:** Diabetic erectile dysfunction, Guipi decoction, RhoA/ROCK, anti-oxidative stress, signaling pathway, diabetes mellitus

## Abstract

**Introduction:**

This study aimed to investigate whether Guipi Decoction ameliorates Diabetic Erectile Dysfunction (DMED) in rats by regulating the RhoA/ROCK signaling pathway.

**Methods:**

36 SD rats were utilized: 6 served as controls, and 30 were induced into DMED models using STZ, confirmed by blood glucose >16.7 mmol/L and a failed erection test. Successfully modeled DMED rats were divided into five groups (n=6/group): Model (saline), Sildenafil (5 mg/kg), and GD Low (0.5 g/mL), Medium (1 g/mL), and High (2 g/mL) dose groups. All groups received corresponding treatments for 4 weeks. Assessments included: body weight, FBG, lipids (TC, TG), erectile function (ICP/MAP ratio), penile NO levels, renal function (BUN, SCr), oxidative stress markers (SOD activity, MDA content), penile histopathology (HE and Masson staining), and mRNA/protein expression levels of RhoA, ROCK1, ROCK2, Caspase-3, p-LIMK2, and p-MYPT1, analyzed *via* qPCR, Western blot, and IHC.

**Results:**

Compared with controls, DMED model rats showed significantly elevated FBG, TG, TC, BUN, SCr, and MDA levels, as well as upregulated expression of RhoA/ROCK pathway-related proteins and Caspase-3 (all *P*<0.01). In contrast, their body weight, ICP/MAP ratio, NO levels, SOD activity, and penile smooth muscle/collagen ratio were significantly reduced (all *P*<0.01). GD treatment exerted significant dose-dependent effects: relative to the Model group, GD increased the ICP/MAP ratio, NO levels, and SOD activity (all *P*<0.01); decreased FBG, weight loss, TC, TG, BUN, SCr, and MDA levels (*P*<0.05 or *P*<0.01); improved erectile function; alleviated penile fibrosis and pathological damage; and downregulated the mRNA and protein expression of RhoA, ROCK1, ROCK2, Caspase-3, p-LIMK2, and p-MYPT1 (*P*<0.05 or *P*<0.01). Higher GD doses showed stronger therapeutic efficacy.

**Discussion:**

The therapeutic effects of GD on DMED may be attributed to its ability to inhibit the RhoA/ROCK signaling pathway, which in turn enhances NO bioavailability, mitigates oxidative stress (as reflected by increased SOD activity and decreased MDA content), and suppresses apoptosis (*via* reduced Caspase-3 expression).

**Conclusion:**

Guipi Decoction effectively improves erectile function and ameliorates penile histopathological lesions in DMED rats, suggesting its potential as a therapeutic agent for DMED through modulation of the RhoA/ROCK signaling pathway.

## 1 INTRODUCTION

Erectile Dysfunction (ED) is one of the common chronic complications in patients with Diabetes Mellitus (DM) [1, 2]. It refers to the inability of a man's penis to achieve or maintain a satisfactory erection during sexual activity, which seriously affects the physical and mental health and quality of life of patients [3]. It is reported [4, 5] that the incidence of ED among patients with DM ranges as high as 37.5% to 66.3%, and the occurrence rate of ED is significantly higher than that in non-diabetic patients, approximately 3.5 times that of the normal population. At present, the pathogenesis of DMED is not fully understood, but studies have shown that DMED is closely associated with oxidative stress, vascular factors, and neurological factors induced by hyperglycemia [4, 6]. It has been reported that the weakening of vascular smooth muscle relaxation in the corpora cavernosa is a key factor in erectile dysfunction [7], and the activation of the Rhoa/ROCK signaling pathway is the main reason for penile weakness due to cavernous smooth muscle contraction [8, 9].

At present, the clinical treatment methods for DMED mainly include surgery, prosthesis implantation, oral phosphodiesterase type 5 (PDE5) inhibitors, negative-pressure suction, *etc.* [10, 11]. Still, all of them have certain adverse reactions and limitations, and the treatment effect is not ideal [12]. Therefore, finding safer and more effective treatments has become a hot topic in this field. With the widespread use of traditional Chinese medicine in the treatment of diabetes and its complications, an increasing number of studies have focused on its potential role in the treatment of DMED [13]. Studies have shown that traditional Chinese medicine can effectively improve erectile function, alleviate clinical symptoms, and improve patients' quality of life [14-16]. In Traditional Chinese Medicine (TCM), ED is primarily categorized as “xiaoke” (consumptive thirst, corresponding to diabetes in modern medicine) and “yangwei” (impotence). Its core pathogenesis is understood to involve spleen Qi deficiency, Qi and blood deficiency, and inadequate nourishment of the penis. Gui Spleen Soup originated from Yan Kuan's “Jisheng Fang” in the Southern Song Dynasty, which is composed of 12 herbs such as Atractylodes macrocephalus, ginseng, astragalus, angelica, boiled licorice, cocoon, Yuanzhi, sour jujube kernel, wood fragrance, longan meat, ginger, jujube, *etc*., and has the effect of strengthening the spleen and invigorating Qi and nourishing the heart and spleen.

Through in-depth research into traditional Chinese medicine, the role of Guipi Decoction in regulating immunity and improving microcirculation has been gradually revealed [17]. Its multi-component effects include lowering blood glucose levels and antioxidant and anti-inflammatory properties, which can improve the metabolic state of diabetic patients and reduce nerve and blood vessel damage, potentially improving therapeutic outcomes for diabetic erectile dysfunction. However, there is still insufficient research on the regulation of the RhoA/ROCK signaling pathway and its effect on DMED. Therefore, this study used the Rhoa/ROCK signaling pathway as a starting point to explore the effects of Guipi Decoction on rats with DMED and to provide new ideas and methods for the prevention and treatment of DMED using traditional Chinese medicine.

## 2 MATERIALS

### 2.1 Animal

A total of 36 SPF-grade SD rats, male, 8 weeks old, with a body weight of (200 ± 20) grams, were provided by Liaoning Changsheng Biotechnology Co., Ltd. All animals were maintained under SPF conditions (20–25°C, 50% ± 20% humidity, 12 h/12 h light–dark cycle) in the experimental animal center, with free access to food and water, and were allowed one week of adaptive feeding.

### 2.2 Medicinal

In Guipi Decoction, there are 9 g White Atractylodes, 6 g Ginseng, 12 g Astragalus, 9 g Angelica, 3 g Fried Licorice, 9 g Poria, 6 g Polygala, 12 g Jujube Seed, 6 g Saussurea, and 12 g Longan Meat, which meets the requirements of the 2020 edition of the Pharmacopoeia of the People’s Republic of China (Appraiser, Unit, Professional Title). Drug Dose Selection Basis: Guipi Decoction was slightly adjusted according to the original formula in the 7^th^ edition of Formula Science (China Traditional Chinese Medicine Press). The following medicinal materials were used: Astragalus membranaceus 20 g (Batch No. 22021401), Ginseng 15 g (Batch No. 22111701), Polygala tenuifolia 8 g (Batch No. 21092801), Atractylodes macrocephala 12 g (Batch No. 22012201), Poria cocos 15 g (Batch No. 22092801), Fried Licorice 15 g (Batch No. 22082603), Saussurea lappa 8 g (Batch No. 21102801), Longan 15 g (Batch No.21102301), and Jujube Seed 15 g (Batch No. 21121301). These slices were purchased from Shijiazhuang Lerentang, along with 6 g of Ginger and 15 g of Jujubes.

The preparation of the medicine followed the Management Standards for Chinese Medicine Decoction Preparation Rooms in Medical Institutions (2006). The herbs were first soaked in water at a 10:1 (herb-to-water) ratio for 1 hour, after which the mixture was brought to a boil, simmered for 40 minutes, and filtered to obtain the first decoction. The remaining herb residue was then re-decocted with water at an 8:1 ratio, boiled, simmered for another 40 minutes, and filtered to obtain the second decoction. The two filtrates were combined and concentrated by heating to a stock solution concentration of 2 g/mL, which was then diluted to working concentrations of 1 g/mL and 0.5 g/mL. All prepared decoctions were stored at 4°C until use.

A preliminary decoction protocol was also conducted for reference. The herbs were soaked in water for approximately 30 minutes, boiled for 30 minutes, and filtered. The residue was then re-boiled with water for another 30 minutes and filtered again. The two decoctions were combined, filtered through gauze, and concentrated to final concentrations of 0.78 g/mL and 1.56 g/mL. Before use, Sildenafil Fumarate Tablets (China National Drug Approval Number H20020527) were dispersed into a suspension with a mass concentration of 5 mg/kg.

### 2.3 Reagent

Triglyceride (TG), Total Cholesterol (TC), Urea Nitrogen (BUN), Serum Creatinine (Scr), Malondialdehyde (MDA), Superoxide Dismutase (SOD), and other biochemical kits were purchased from the Nanjing Institute of Bioengineering. The following primary antibodies were obtained from Wuhan San Ying Biotechnology Co., Ltd.: Ras Homology Gene Family Member A (RhoA), Rho-Related Coiled- Coil Helical Protein Kinase 1 (ROCK1), Rho-Related Coiled-Coil Helical Protein Kinase 2 (ROCK2), Caspase-3 (Caspase-3), and Glyceraldehyde-3-Phosphate Dehydrogenase (GAPDH), with item numbers 10749-1-AP, 21850-1-AP, 21645-1-AP, 25128-1-AP, and 10494-1-AP, respectively. Primary antibodies against Phosphorylated LIM Kinase 2 (p-LIMK2) and Phospho-Myosin Phosphatase Target Subunit 1 (p-MYPT1) were purchased from Abcam (item numbers ab38499 and ab59235).

### 2.4 Instrument

The SpectraMax i3 multi-functional full-wavelength microplate reader (Meigu Molecular Instrument Co., Ltd.), the ST16R high-speed refrigerated centrifuge (Thermo, USA), the T100 thermal cycling PCR instrument (Bio-Rad, USA), the WIX-miniPRO4 vertical electrophoresis tank, and the WIX-mini BLOT transfer tank (Beijing Weixs Company) were used in this study.

## 3 METHODS

### 3.1 DMED Rat Model Preparation

Healthy male SD rats were randomly divided into six groups: with 8 rats in each group: the control group (Control group), the DMED model group (Model group), and the Sildenafil group, as well as the low-, medium-, and high-dose GD groups. After 1 week of free feeding and adaptive rearing, rats in the Control group were maintained on a standard diet and normal feeding conditions until the end of the experiment. For rats in the DMED Modeling group, Streptozotocin (STZ) was intraperitoneally injected at a dose of 40 mg/kg into the left lower abdomen to induce diabetes. At 72 hours post-injection, blood was collected from the tail vein to measure blood glucose levels. Two weeks after STZ injection, rats with confirmed diabetes (DM rats) were transferred to an observation box. The observation room was set to dim lighting (sufficient only for visual observation) and maintained in a quiet environment. Following a 10-minute acclimatization period, 100 μg/kg of Apomorphine (APO) solution (containing 5 mg APO, 250 mg vitamin C, and 500 mL sterile normal saline) was injected into the loose skin of the rats’ necks. A 30-minute observation period was then conducted, during which the occurrence and frequency of penile erections were recorded.

In this experiment, penile erection was defined by the presence of four morphological signs: penile enlargement, penile congestion, foreskin retraction, and exposure of the penile glans. An erection is considered positive if it occurs at least once; otherwise, it is considered negative. Rats with a blood glucose level > 16.7 mmol/L and a negative erection test were classified as DMED rats, which were then assigned to the DMED Positive group and subsequent Guipi Decoction intervention groups for further experiments. This study was approved by the Ethics Committee of the Second Affiliated Hospital of Liaoning University of Traditional Chinese Medicine, with approval number: AEC01002-RS01.

### 3.2 Experimental Intervention and Grouping

The 30 DMED rats that were successfully modeled were divided into 5 groups according to a random number table: model group, Sildenafil group, and low-, medium-, and high-dose GD groups. According to the dosing conversion method for humans and experimental animals, sildenafil was administered at 5 mg/kg (in saline) *via* gavage to the sildenafil group. The GD low, medium, and high doses (0.5 g/mL, 1 g/mL, and 2 g/mL, respectively) were administered *via* gavage. The control and model groups received an equal volume of saline daily for 4 weeks.

### 3.3 General Condition and Body Weight of Rats

The diet, water intake, hair luster, activity, mental state, and urine and feces of the rats were observed daily. At the end of the experiment, the rats in each group were fasted for 12 hours, and their body mass was recorded.

### 3.4 Biochemical Analysis of Fasting Blood Glucose and Lipids

Blood was collected from the tail vein 24 hours after the end of administration. After standing, the samples were centrifuged at 3000 rpm for 10 minutes, and the supernatant was collected to measure FBG, TC, and TG levels using a fully automatic biochemical analyzer.

### 3.5 Multi-Electrode Assessment of Rat Penile Erection Function

After blood collection, 10% chloral hydrate was injected intraperitoneally to anesthetize the rat. After fixation, the right common carotid artery was separated and punctured with a 22-G needle filled with 250 U/mL heparin solution and connected to the multichannel electrophysiology instrument. Expose the midline incision of the rat's lower abdomen, free the corpus cavernosum nerve, and cut open the skin covering the base of the penis to expose both penile roots. Separate the base of the penis and insert a 22-G needle filled with 250 U/mL heparin solution into the left corpus cavernosum, connecting it to a pressure monitoring system. Hook the silver wire electrodes of the pressure monitoring system onto the corpus cavernosum nerve on the other side for continuous single stimulation. The stimulation parameters are set at 5 ms, 5 V, 15 Hz, with a duration of 1 minute and an interval of 5 minutes. Record the Intracavernous Pressure (ICP) in the corpus cavernosum. The operator responsible for invasive ICP/MAP (mean arterial pressure) measurements was unaware of the rat group assignments, with all rats identified only by code; pressure data were recorded and stored under codes before post-hoc group unblinding.

### 3.6 NO Levels in Rat Penile Corpus Cavernosum (Nitrate Reductase Method)

The rat penile corpora cavernosa tissue was homogenized using a homogenizer, and the supernatant was collected after centrifugation at 4,000 rpm for 10 minutes. The rat NO level was measured using the NO determination kit (nitric acid reductase method), and all procedures were performed strictly according to the kit instructions.

### 3.7 Renal Function Assessment in Rats

Serum stored as described in section 2.4 was used to measure the levels of SCr and BUN according to the kit instructions.

### 3.8 SOD and MDA Levels in Rat Penile Tissue

The tissue of the penile corpus cavernosum was homogenized, and the activity of SOD and the content of MDA were measured according to the kit instructions.

### 3.9 H&E Staining of Rat Penile Corpus Cavernosum

The rat penile corpora cavernosa tissue was fixed in 10% formalin, dehydrated in a graded ethanol series, and embedded in paraffin. The tissue sections (4 μm thick) were mounted on slides, dewaxed with xylene, and sequentially stained with hematoxylin and eosin. The sections were then dehydrated with a gradient ethanol solution, cleared with xylene, and sealed with neutral resin. The pathological changes in the penile tissue were observed under an optical microscope.

### 3.10 Masson’s Staining of Penile Corpus Cavernosum Fibrosis

In accordance with the Masson method, the newly extracted penile corpus cavernosa was washed with normal saline, and the middle and posterior segments were fixed in 4% polyformaldehyde solution. After dehydration and clearing with ethanol and xylene, it was embedded in paraffin, cut into 5 μm-thick sections, and stained with Masson. The Olympus microscopic imaging system was used for observation and photography, and the area ratio of penile corpus cavernosa smooth muscle tissue (red) to collagen tissue (blue) was calculated using ImageJ analysis software to reflect the degree of penile corpus cavernosa fibrosis.

### 3.11 Real-Time PCR of RhoA, ROCK1/2, and Caspase-3 mRNA in Rat Corpus Cavernosum

The total RNA from the penile corpus cavernosa tissue was extracted using an ice box. The reaction mixture was incubated at 42°C for 2 minutes using a reverse transcription kit. The reaction mixture was added and incubated at 42°C for 15 minutes, followed by a 5-minute incubation at 85°C to reverse-transcribe to cDNA. The cDNA was amplified using SYBR Green, with the following conditions: 95°C for 30 seconds pre-denaturation, 95°C for 5 seconds denaturation, and 60°C for 30 seconds annealing, for a total of 45 cycles. The 2-ΔΔCt method was used for analysis, and the PCR primers were designed and synthesized by Shengong Biological Engineering (Shanghai) Co., Ltd. The primer sequences are presented in Table **1**.

### 3.12 Western Blot of RhoA, ROCK1/2, p-LIMK2, p-MYPT1, and Caspase-3 in Rat Corpus Cavernosum

Tissue protein was extracted, and the protein concentration was determined using a BCA kit prior to loading. Electrophoresis was performed at 160 V. Once the target protein reached the bottom of the gel, it was transferred to a membrane at 400 mA. The membrane was then blocked with a quick blocking solution for 10 minutes, followed by a wash with TBST. Working solutions of RhoA (1:4,000), ROCK1 (1:25,000), ROCK2 (1:2,000), p-LIMK2 (1:750), p-MYPT1 (1:750), and Caspase-3 (1:1,000) were added, and the membrane was incubated overnight at 4°C. The following day, the membrane was washed with TBST, and rabbit anti- mouse (1:60,000) and mouse anti-mouse (1:5,000) secondary antibodies were applied. The membrane was incubated for 1.5 hours, washed again, and developed. The grayscale values were analyzed using ImageJ.

### 3.13 Immunohistochemical Detection of RhoA, ROCK1, and Caspase-3

The rat penile corpora cavernosa tissue, fixed in 4% polyformaldehyde, underwent dehydration, paraffin embedding, and slicing (5 μm thick). The sections were routinely dehydrated at 60°C. After the sections were placed in a gradient of alcohol and water, they were incubated in 3% H_2_O_2_ at room temperature for 10 minutes to inactivate endogenous peroxidase. The sections were then washed with PBS and placed in sodium citrate buffer. The buffer was heated to boiling in a microwave oven and then cooled off. This process was repeated twice, with a 10-minute interval between each heating, and the sections were naturally cooled to room temperature to complete the antigen repair. After PBS washing, add BSA blocking solution and incubate at room temperature for 15 minutes. Add rabbit anti-rat antibody (4°C) and incubate overnight. Wash with PBS, add the secondary antibody working solution, and incubate at room temperature for 20 minutes. Perform DAB staining, rinse with tap water, and then stain with hematoxylin. Dehydrate and dry using a graded alcohol series, clear with xylene, and mount with neutral resin. The Mean Optical Density (MOD) was analyzed using Image J software (Version 1.8.0). Five non-overlapping fields were randomly selected for imaging under 400× magnification (with unified light source and exposure parameters). Images were converted to grayscale, and the background was subtracted (rolling ball radius = 50 pixels). The cytoplasm of smooth muscle cells was delineated as the region of interest (ROI). Integrated Optical Density (IOD) of the ROI was measured, and MOD was calculated as “MOD = IOD/ROI area”. The average value of 5 fields per rat was obtained, and data in each group were expressed as the mean ± standard deviation of 6 rats.

### 3.14 Statistical Methods

SPSS 23.0 software was used for analysis. Measurement data were described as Mean (SD) when normally distributed and as median (IQR) when not. When the measurement data were normally distributed, variance analysis was used; when they were not normally distributed, and variance was not equal, the Wilcoxon nonparametric method was used, followed by Tukey’s Honestly Significant Difference (HSD) test for post-hoc pairwise comparisons (to identify specific differences between groups). Data were expressed as mean ± standard deviation (x̄ ± s), and *P* <0.05 indicated that the difference was statistically significant.

## 4 RESULTS

### 4.1 Blood Glucose and Body Weight Changes in Each Rat Group

As presented in Table **2**, compared with the control group, the model group rats had significantly increased blood glucose and significantly decreased body weight before and after treatment (*P*<0.01). Compared with the model group, there was no significant difference in blood glucose or body weight in rats across treatment groups before treatment (*P*>0.05). After treatment, blood glucose and body weight decreased in rats across all treatment groups. Body weight of rats increased in the sildenafil group (*P*<0.01), and there was no significant difference in blood glucose of rats in the sildenafil group (*P*>0.05).

### 4.2 Comparison of ICP, MAP, and ICP/MAP in Each Group of Rats

Compared with the control group, the ICP, MAP, and ICP/MAP of the model group rats were significantly reduced (*P*<0.01), while the MAP difference was not statistically significant (*P*>0.05). Compared with the model group, the ICP and ICP/MAP in the rats of each drug administration group were significantly increased (*P*<0.01), whereas the MAP difference was not statistically significant (*P*>0.05) (Table **3**).

### 4.3 NO Levels in the Penile Corpus Cavernosum of Each Rat Group

The NO level in the corpora cavernosa tissue of rats was significantly different between groups (*P*<0.01). Compared with the control group, the NO level in the corpora cavernosa tissue of model rats was significantly reduced (*P*<0.01). Compared with the model group, NO levels in the corpora cavernosa tissue of the sildenafil group and the Guipi Decoction groups at low, medium, and high doses were significantly increased (*P*<0.05) (Table **4**).

### 4.4 Comparison of TG, TC, BUN, and SCr in Rats

Compared with the blank group, the TG, TC, BUN and SCr levels of the model group rats were significantly increased (*P*<0.01); compared with the model group, the TG, TC, BUN and SCr levels of the sildenafil group and the low, medium and high doses of Guipi Decoction group rats were significantly decreased (*P*<0.01) (Table **5**).

### 4.5 Determination of SOD and MDA Content in Rat Penile Tissue

As presented in Table **6**, compared with the normal group, SOD activity in penile tissue of the model group was significantly reduced (*P* <0.01), and MDA content was significantly increased (*P* <0.01). In contrast, the SOD activity in the penile tissue of the rats in the low, medium, and high doses of Guipi Decoction and the sildenafil group was significantly increased (*P* <0.01), and the MDA content was significantly decreased (*P* <0.01).

### 4.6 Histopathological Examination of Rat Corpus Cavernosum *via* Hematoxylin-Eosin Staining

As shown in Fig. (**1**), compared with the intact histological structure of the control group, the model group exhibited significant pathological alterations, including disordered smooth muscle cell arrangement, thickened vascular walls, luminal stenosis, and inflammatory cell infiltration. These alterations were ameliorated in various dosage groups of Guipi Decoction and the Sildenafil group, which demonstrated improved structural integrity, relatively organized arrangement of smooth muscle fibers, reduced vascular wall thickness, improved luminal stenosis, and diminished inflammatory infiltration.

### 4.7 Masson's Trichrome Staining of Rat Corpus Cavernosum Fibrosis

As shown in Fig. (**2**), the corpus cavernosum tissue of the normal control group exhibited an appropriate area ratio between smooth muscle tissue (stained red) and collagenous tissue (stained blue). In the model group, a significant increase in collagenous tissue area was observed, accompanied by a marked reduction in the smooth muscle-to-collagen area ratio (*P*<0.01), indicating more severe cavernous fibrosis. Compared with the model group, the medium-dose Guipi Decoction group and sildenafil group demonstrated significantly decreased collagenous tissue accumulation (*P*<0.05), with corresponding elevation in the smooth muscle-to-collagen area ratio and attenuation of fibrotic changes in the corpus cavernosum (Table **7**).

### 4.8 Real-Time qPCR of Rat Corpus Cavernosum Genes

Compared to the normal group, the model group showed significantly increased mRNA expression of RhoA, ROCK1, and Caspase-3 (*P* < 0.01), as well as significantly elevated ROCK2 expression (*P* < 0.05) in corpus cavernosum tissues. The low-dose Guipi Decoction group significantly reduced the expression of RhoA, ROCK1, and Caspase-3 (*P* < 0.01), while the reduction in ROCK2 was not statistically significant (*P* > 0.05). In contrast, the medium- dose and high-dose Guipi Decoction groups, and the sildenafil control group, all significantly downregulated the mRNA expression of all four genes (RhoA, ROCK1, ROCK2, Caspase-3; *P* < 0.01) compared to the model group, with the high-dose Guipi Decoction group exhibiting the most pronounced inhibitory effect (Table **8**).

### 4.9 Western Blot of Rat Corpus Cavernosum Proteins

The Western blot results demonstrated that compared with the normal control group, the model group exhibited significantly elevated protein expression levels of RhoA, ROCK1, ROCK2, p-LIMK2, p-MYPT1, and Caspase-3 in corpus cavernosum tissues (*P* < 0.01) (Table **9**). When compared with the model group, the low-dose Guipi Decoction group showed significantly reduced protein expression of RhoA (*P* < 0.05), ROCK1 (*P* < 0.01), ROCK2 (*P* < 0.01), and Caspase-3 (*P* < 0.05). In contrast, the reduction in p-LIMK2 expression did not reach statistical significance (*P* > 0.05). Notably, the medium- and high-dose Guipi Decoction groups, and the sildenafil control group, all demonstrated significant downregulation of RhoA, ROCK1, ROCK2, p-LIMK2, p-MYPT1, and Caspase-3 protein expression (*P* < 0.01) compared with the model group. Representative immunoblots are shown in Fig. (**3**).

### 4.10 Immunohistochemistry of Rat Corpus Cavernosum Proteins

As shown in Figs. (**4A**-**F**), immunohistochemical staining revealed significantly elevated protein expression levels of RhoA, ROCK1, and Caspase-3 in the corpus cavernosum of model group rats compared with the control group (*P* < 0.01), as evidenced by increased mean optical density (MOD) values. Compared with the model group, the medium- and high-dose Guipi Decoction groups, as well as the sildenafil control group, exhibited significantly reduced protein expression of RhoA, ROCK1, and Caspase-3 (*P* < 0.01), accompanied by decreased MOD values. At the same time, the reduction in the low-dose group of Guipi Decoction was not statistically significant (*P* > 0.05). These findings suggest that Guipi Decoction exerts dose-dependent regulatory effects on cavernous tissue protein expression patterns (Table **10**).

## 5 DISCUSSION

Diabetes mellitus is a metabolic disease characterized by glucose metabolism disorders [18], resulting in a persistent state of hyperglycemia that leads to a series of complications, among which erectile dysfunction is common [4, 19, 20]. In recent years, research on diabetic erectile dysfunction (DMED) has deepened, and the efficacy of traditional Chinese medicine in its treatment has been increasingly confirmed [21]. Traditional Chinese medicine posits that deficiency of heart-Qi, spleen-Qi, and Qi-blood prevents proper circulation to the penis, resulting in flaccidity [22]. As stated in Jing Yue Quan Shu Impotence: “... illness and Yangming pulse, and Shui Gu's sea of qi and blood will suffer. Deficiency of qi and blood leads to depression of Yangdao” [23].

Based on symptom differentiation, Guipi Decoction is selected for treatment [24]. In this formula, Astragalus membranaceus and Longan pulp serve as the monarch herbs, invigorating the spleen and Qi while regulating the heart and blood. Ginseng, Atractylodes macrocephala, and Astragalus membranaceus act as minister herbs to tonify the spleen [25], while *Angelica sinensis* and *Ziziphus jujuba* nourish the heart and blood. *Radix Aucklandiae* serves as an assistant to regulate Qi and strengthen the spleen, ensuring the herbs are supplemented without stagnation. Poria and Polygalae assist in nourishing the heart and calming the mind, with Ginger, Licorice, and Jujube completing the formula. Therefore, Buyi Guipi Decoction has a strong theoretical basis for treating diabetic erectile dysfunction [26].

In this study, the pressure monitoring system showed that, compared with the control group, the model group exhibited decreased erectile function, confirming the success of the model. After intervention with Guipi Decoction, erectile function in DMED rats was significantly improved, effectively alleviating diabetic erectile dysfunction and reducing pathological changes and fibrosis in the corpus cavernosum, demonstrating a favorable therapeutic effect.

In recent years, oxidative stress induced by a hyperglycemic environment has been identified as a key mechanism driving the development of Diabetic Erectile Dysfunction (DEMD). As critical components of the corpus cavernosum, Cavernous Smooth Muscle Cells (CCSMCs) and endothelial cells play an essential role in both the initiation and maintenance of penile erection [9]. Nitric Oxide (NO), a vasorelaxant produced by endothelial cells, regulates the contraction and relaxation of smooth muscle cells [27]. Under hyperglycemic conditions, the activity of free radical-scavenging enzymes (*e.g*., superoxide dismutase, SOD) is suppressed, leading to increased production of Reactive Oxygen Species (ROS) and elevated levels of lipid peroxides such as Malondialdehyde (MDA), collectively intensifying oxidative stress [28]. Activation of the cysteine protease Caspase-3 triggers apoptosis in both endothelial cells and CCSMCs [29]. This apoptotic process, in turn, reduces NO release, impairs the contractile and relaxant functions of penile smooth muscle [30], and ultimately contributes to the development of DEMD [31]. In the present study, compared with the control group, the DEMD model group exhibited decreased SOD activity, increased MDA and Caspase-3 levels, and reduced NO content. These findings indicate that DEMD rats have impaired antioxidant capacity, accompanied by heightened oxidative stress and cellular apoptosis. Following intervention with Guipi Decoction, SOD activity and NO levels were significantly elevated, while MDA and Caspase-3 levels were reduced. These results suggest that Guipi Decoction effectively mitigates oxidative stress and alleviates cellular apoptosis in rats with DEMD.

In recent years, studies have found that the RhoA/ROCK pathway is upregulated in DEMD rats. This pathway can affect the contraction and relaxation of penile smooth muscle cells by activating a series of downstream molecules. RhoA belongs to the Rho GTPase subclass of the Ras superfamily and possesses GTPase activity. It can switch between GTP-bound and GDP-bound states, acting as a molecular switch to regulate intracellular signal transduction. After RhoA activation, it transitions from the binding state to the active state. It binds to the Rho-binding domain of ROCK, leading to the release of the kinase domain and the phosphorylation of multiple amino acid residues, thereby activating ROCK [32]. Rho-Associated Coiled-Coil Containing Kinases (ROCK) are serine-threonine kinases with two isoforms, ROCK1 and ROCK2. MYPT1 and LIMK are key effector molecules downstream of ROCK. The phosphorylation status of MYPT-1-specific residue regions correlates with MLCP expression inhibition [33]. Upon ROCK activation, MYPT-1 on MLCP is phosphorylated, inhibiting MLCP activity and promoting smooth muscle cell contraction [34]. LIMK, as a key enzyme for cofilin phosphorylation, can promote the formation of actin. Research has found that ROCK can promote smooth muscle contraction by activating LIMK [35]. Li *et al*. [36] found that the expression of the RhoA/ROCK signaling pathway and the contraction of smooth muscle cells increased in the penis tissue of diabetic rats, resulting in erectile dysfunction. Apocynum venetum could significantly reduce the expression of RhoA, ROCK1, and p-MYPT1 in the penis tissue of diabetic rats, and improve erectile dysfunction in diabetic rats. This study found that after intervention with Guipi Decoction, DEMD rats downregulated the mRNA and protein expression of RhoA, ROCK1, and ROCK2, while reducing the protein levels of p-LIMK2 and p-MYPT1.

## 6 STUDY LIMITATIONS

This study has several limitations: the exclusive use of male SD rats limits the applicability of the findings to females and other species. The research primarily focused on the RhoA/ROCK pathway, while diabetic erectile dysfunction involves complex, multifactorial mechanisms that remain insufficiently explored. Future studies should incorporate diverse animal models, investigate broader molecular networks, isolate bioactive compounds, and evaluate chronic dosing regimens [37].

## CONCLUSION

To sum up, the results of this study found that Guipi Decoction may enhance the erectile capacity of rats with diabetes induced erectile dysfunction and alleviate the pathological damage and fibrosis process of the cavernous body of the penis by regulating the RhoA/ROCK signal pathway. Its mechanism of action may involve increasing NO concentration, resisting oxidative stress, and inhibiting cell apoptosis. However, this experiment still has certain limitations. The pathological damage of the corpus cavernosum of the penis involves multiple pathways, and the specific mechanism is not yet clear. Whether Guipi Decoction can alleviate the pathological damage of the corpus cavernosum of the penis through mutual regulation of multiple pathways still needs further verification.

## Figures and Tables

**Fig. (1):**
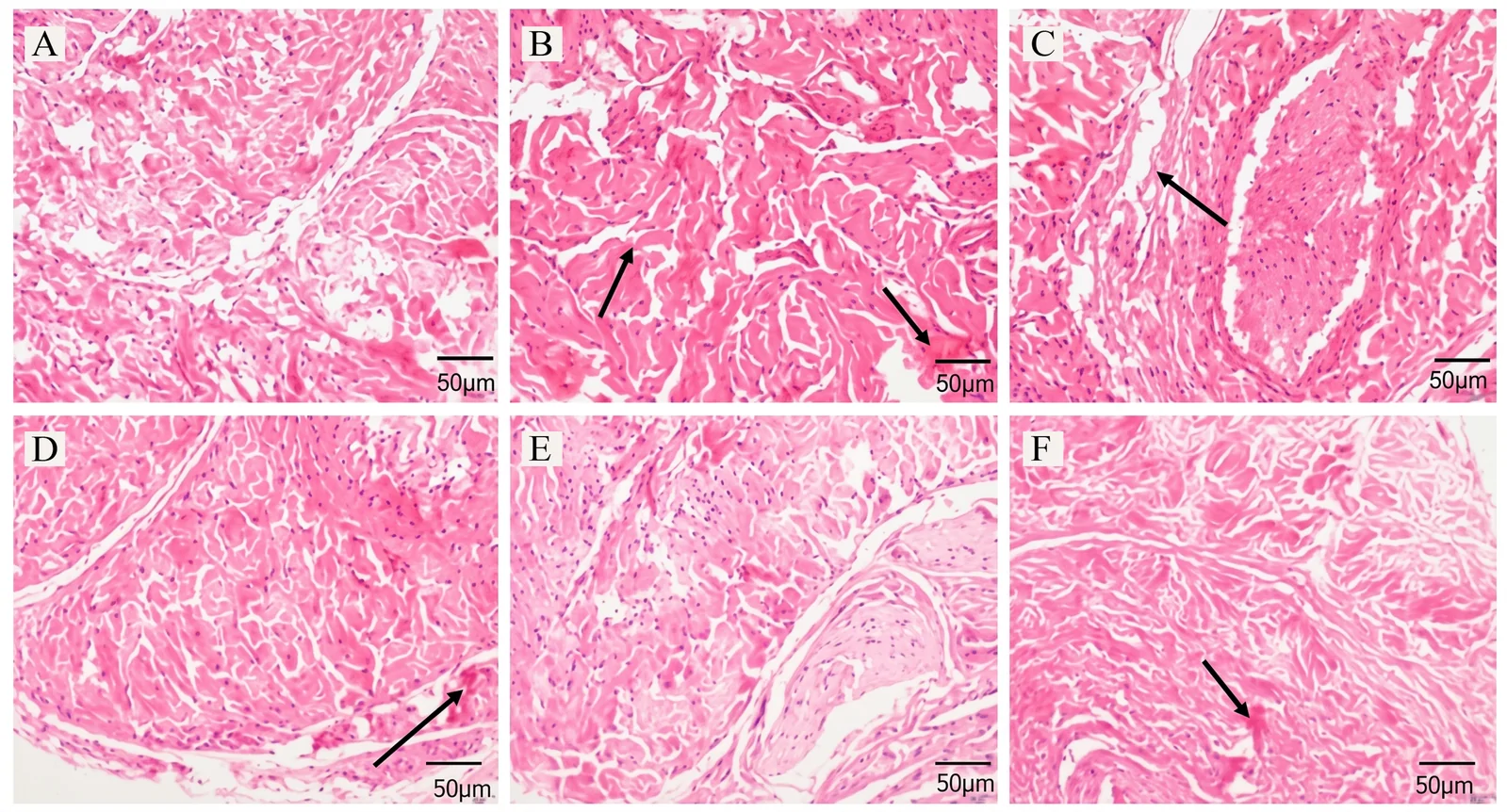
HE observed the corpus cavernosum pathology in rats (HE ×200). **(A-F)** represent control, model, low, medium, and high doses of Guipi Decoction, and the sildenafil group, respectively.

**Fig. (2):**
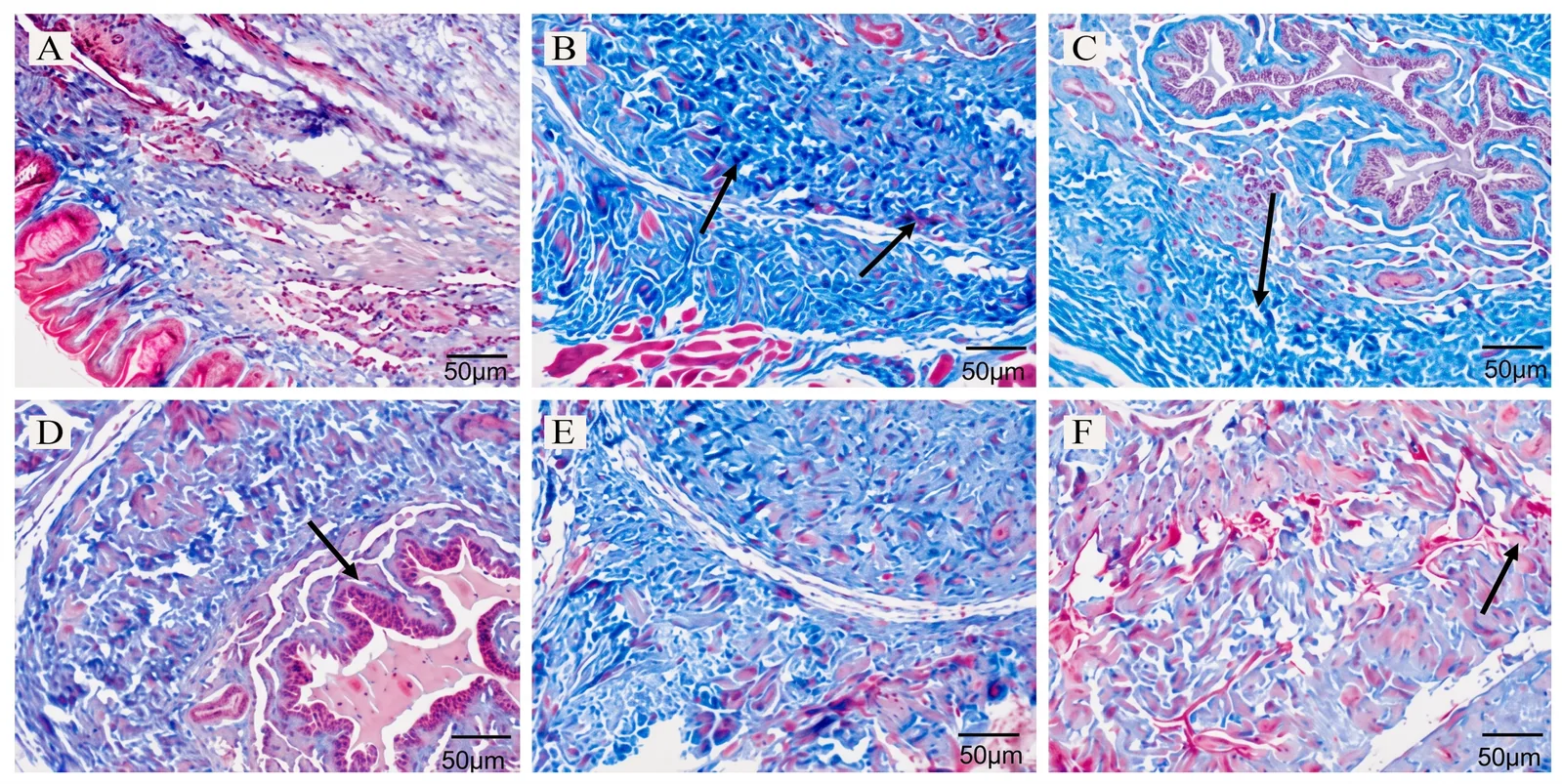
Rat penis cavernosum fibrosis was observed by the Masson method (Masson ×200). (**A-F**) represent control, model, low, medium, and high doses of Guipi Decoction, and the sildenafil group, respectively.

**Fig. (3):**
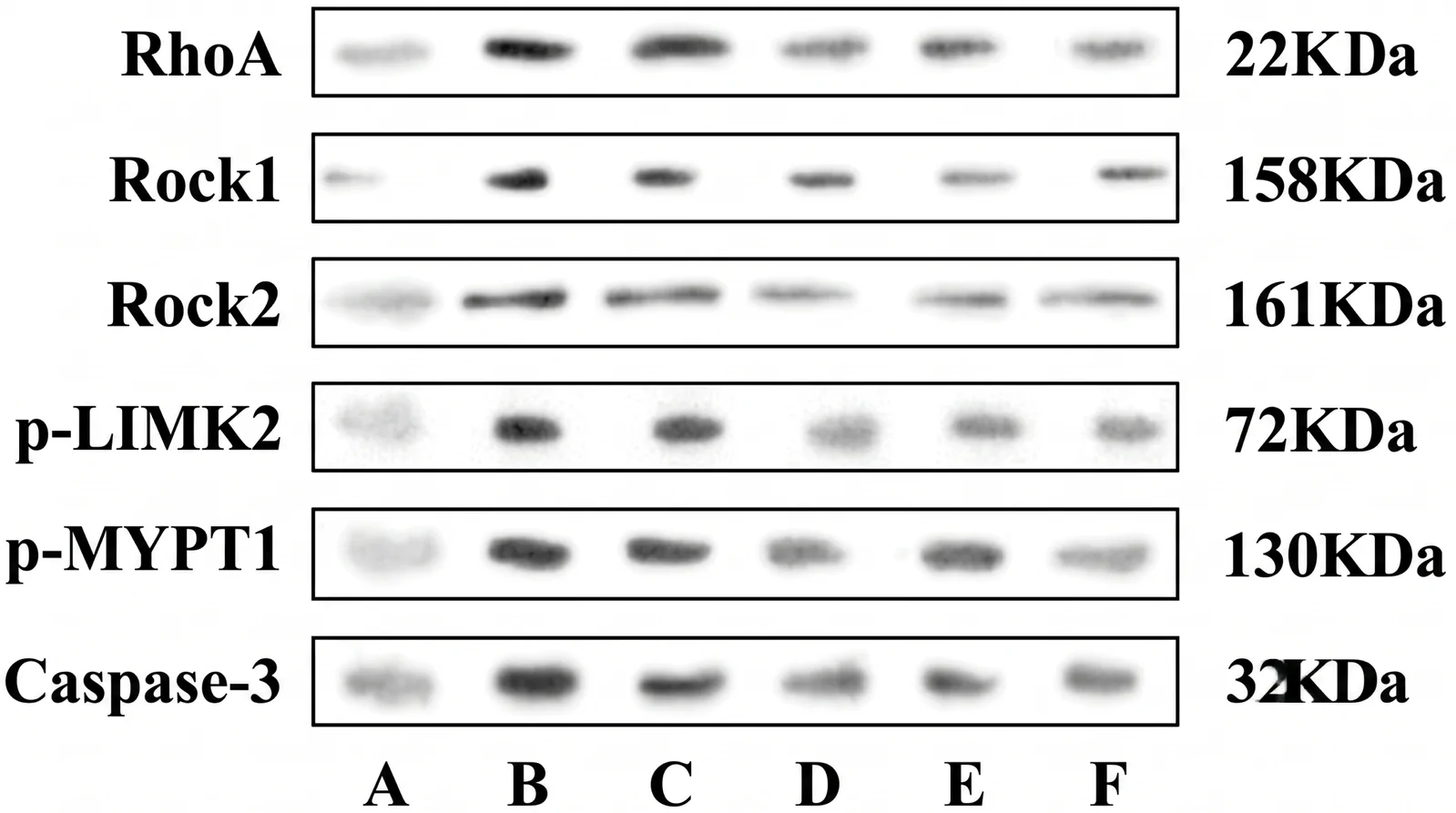
WB Detection of the corpus cavernosum tissue of the rat penis. RhoA, ROCK1, ROCK2, p-LIMK 2, p-MYPT 1, and Caspase-3 showed protein expression. **(A-F)** represent control, model, low, medium, and high doses of Guipi Decoction, and the sildenafil group,respectively

**Fig. (4):**
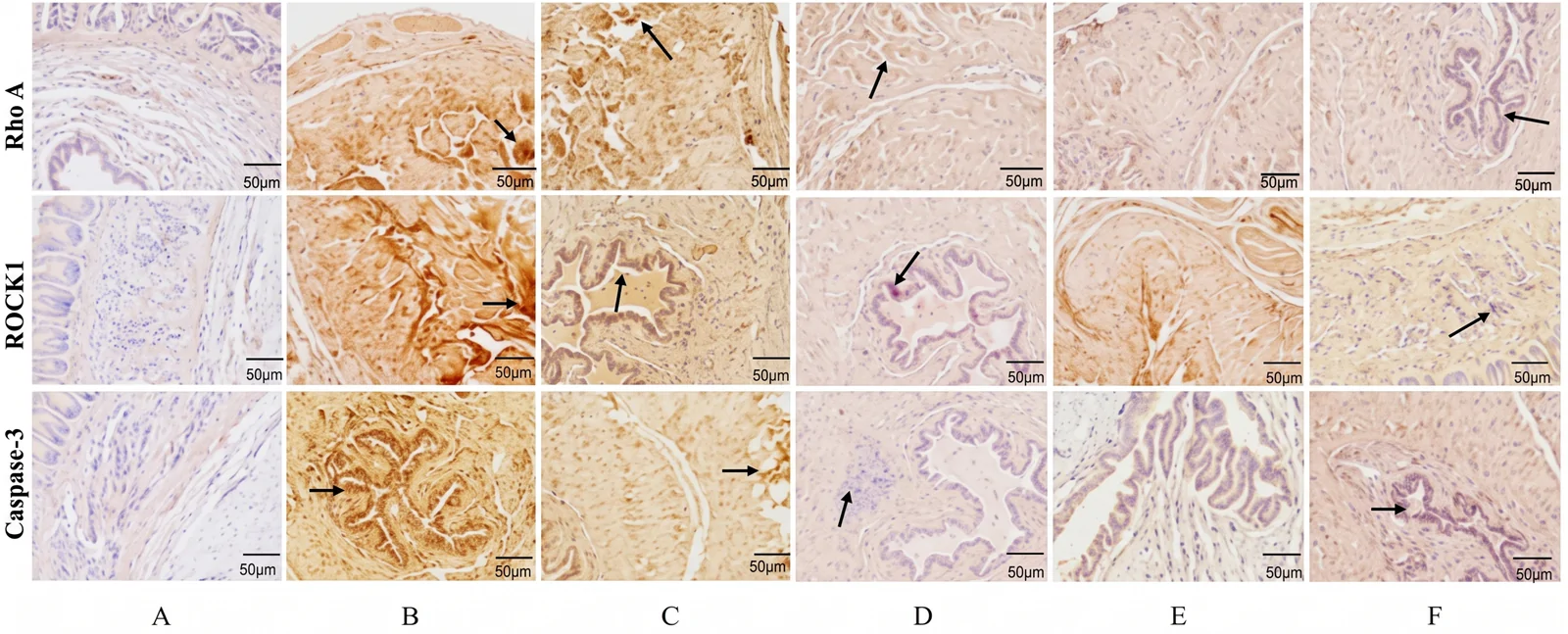
Immunohistochemical detection of protein expression of rat corpus cavernosum tissue (400×). (**A**-**F**) represent control, model, low, medium, and high doses of Guipi Decoction, and the sildenafil group, respectively.

**Table 1:** Primer sequences for PCR.

Gene	Primer Sequence	Length/bp
RhoA	Forward primer: AGCTTGTGGTAAGACATGCTTG	138
-	Reverse primer:GTGTCCCATAAAGCCAACTCTAC	-
ROCK1	Forward primer: GACTGGGGACAGTTTTGAGAC	104
-	Reverse primer:GGGCATCCAATCCATCCAGC	-
ROCK2	Forward primer: TTGGTTCGTCATAAGGCATCAC	124
-	Reverse primer: TGTTGGCAAAGGCCATAATATCT	-
Caspase-3	Forward primer:ATGGAGAACAACAAAACCTCAGT	74
-	Reverse primer:TTGCTCCCATGTATGGTCTTTAC	-
GAPDH	Forward primer: AGGTCGGTGTGAACGGATTTG	123
-	Reverse primer: TGTAGACCATGTAGTTGAGGTCA	-

**Table 2:** Comparison of blood glucose and body weight before and after treatment of rats (x ± s).

Group	n	Blood Sugar /(mmol/L)		Avoirdupois /g	
		Pretherapy	Post-treatment	Pretherapy	Post-treatment
Control	6	4. 77±0.41	4. 63±0.62	350. 41±20.85	439. 72±36.65
Model	6	19. 38±3.22^##^	23. 49±6.83^##^	314. 54±28.96^##^	290. 09±42.39^##^
Low-dose group of Gui Pi Decoction	6	19. 68±4.14	15. 13±2.80**	318. 87±31.07	301. 33±39.72**
Dose group of Gui pi Decoction	6	20. 34±3.85	12. 99±3.64**	324. 33±29.18	318. 03±54.38**
High-dose group of Gui pi Decoction	6	20. 19±3.82	11. 02±3.40**	317. 75±20.22	315. 75±31.22**
Sildenafil group	6	-	24. 21±3.96	325. 16±20.48	286. 91±43.63**
*F*	-	21. 81	23. 86	1. 554	10. 47

**Note:** Compared with the control, ^##^*P*<0.01; compared with the model, **P<0.01.

**Table 3:** Comparison of ICP, MAP, and ICP / MAP in rats in each group (x ± s, n=6).

Group	n	ICP/mmHg	MAP/mmHg	ICP/MAP/%
Control	6	90. 17±6.47	130. 57±2.54	66. 76±2.14
Model	6	19. 83±2.79^##^	128. 26±2.58	13. 46±2.86^##^
Low-dose group of Gui Pi Decoction	6	28. 33±3.55**	131. 98±3.01	19. 63±2.82**
Dose group of Gui Pi Decoction	6	39. 67±2.20**	144. 07±2.40	-
High-dose group of Gui Pi Decoction	6	49. 83±3.93**	136. 49±2.18	34. 88±2.13**
Sildenafil group	6	67. 00±2.61**	130. 71±3.11	49. 37±2.25**
*F*	-	274. 60	26. 32	411. 50

**Note:** Compared with the control, ^##^*P*<0.01; compared with the model, **P<0.01.

**Table 4:** Effect of Guipi Decoction on NO levels of penile cavernosum in each group of rats (x ± s, n=6).

Group	Dosage	NO/μmol·L^-1^
Control	/	-
Model	/	2. 52±0.50^##^
Low-dose group of Guipi Decoction	0.5 g /mL	3. 16±0.63**
Dose group of Guipi Decoction	1 g /mL	5. 33±0.39**
High-dose group of Guipi Decoction	2 g /mL	5. 87±0.43**
Sildenafil group	5 mg/kg	5. 37±0.48**
*F*	-	42. 02

**Note:** Compared with the control, ^##^*P*<0.01; compared with the model, **P<0.01.

**Table 5:** Comparison of TG, TC, BUN, and SCr before and after treatment in each group (x ± s).

Group	Dosage	TG/mmol·L^-1^	TC/mmol·L^-1^	BUN/mmol·L^-1^	SCr/μmol·L^-1^
Control	/	0. 44±0.04	1. 24±0.08	5. 37±0.81	-
Model	/	0.92±0.05^##^	2. 53±0.16^##^	15. 27±0.69^##^	34. 09±1.28^##^
Low-dose group of Guipi Decoction	0.5 g /mL	0.76±0.03**	1. 57±0.06**	12. 17±0.97**	30. 33±1.44*
Dose group of Guipi Decoction	1 g /mL	0.63±0.04**	1. 62±0.09**	9. 30±1.31**	27. 87±1.17**
High-dose group of Guipi Decoction	2 g /mL	0.57±0.05**	1. 51±0.04**	7. 32±1.28**	25. 39±1.34**
Sildenafil group	5 mg/kg	0.53±0.04**	1. 49±0.08**	6. 63±0.83**	23. 19±2.13**
*F*	-	97. 90	135. 50	90. 16	73. 84

**Note:** Compared with the control, ^##^*P*<0.01; compared with the model, **P<0.01.

**Table 6:** SOD and MDA content determination of rat penile tissue.

Group	Dosage	SOD/mmol·L^-1^	MDA/U·L^-1^
Control	/	23. 75±0.20	13. 24±0.62
Model	/	16. 01±0.79^##^	-
Low-dose group of Guipi Decoction	0.5 g /mL	18. 39±0.73**	16. 06±0.48**
Dose group of Guipi Decoction	1 g /mL	18. 89±0.40**	14. 83±0.23**
High-dose group of Guipi Decoction	2 g /mL	20. 06±0.15**	14. 18±0.52**
Sildenafil group	5 mg/kg	20. 51±0.30**	13. 90±0.48**
*F*	-	220. 70	90. 66

**Note:** Compared with the control, ^##^*P*<0.01; compared with the model, **P<0.01.

**Table 7:** Observation of the area of smooth muscle tissue (red) and collagen tissue (blue) in the corpus cavernosum of the rat penis by Masson's method.

Group	Dosage	Red/Blue
Control	/	11. 00±1.40
Model	/	0. 17±0.07^##^
Low-dose group of Guipi Decoction	0.5 g /mL	0. 33±0.086
Dose group of Guipi Decoction	1 g /mL	5. 97±0.01*
High-dose group of Guipi Decoction	2 g /mL	0. 26±0.02
Sildenafil group	5 mg/kg	3. 96±1.86*
*F*	-	397. 40

**Note:** Compared with the control, ^##^*P*<0.01; compared with the model, *P<0.05.

**Table 8:** The mRNA expression of RhoA, Rock1, Rock2, and Caspase-3 in the rat corpus cavernosum penis was detected by PCR.

Group	Dosage	RhoA	Rock1	Rock2	Caspase-3
Control	/	1	1	1	1
Model	/	2. 16±0.07^##^	3. 50±0.73^##^	4. 37±1.66^##^	3. 67±0.29^##^
Low-dose group of Guipi Decoction	0.5 g /mL	1. 52±0.07**	1. 15±0.20**	2. 17±1.02	1. 79±0.25**
Dose group of Guipi Decoction	1 g /mL	1.41±0.22**	1.29±0.18**	0.73±0.41**	0.56±0.10**
High-dose group of Guipi Decoction	2 g /mL	0.59±0.01**	0.43±0.12**	0.35±0.18**	0 28±0.04**
Sildenafil group	5 mg/kg	0.52±0.09**	1.13±0.02**	0.40±0.22**	0.33±0.11**

**Note:** Compared with the control, ^##^*P*<0.01; compared with the model, **P<0.01.

**Table 9:** WB detection of the corpus cavernosum tissue of the rat penis. RhoA, ROCK1, ROCK2, p-LIMK 2, p-MYPT 1, and Caspase-3 showed protein expression.

Group	Dosage	RhoA/ GADPH	ROCK1/ GADPH	ROCK2/ GADPH	p-LIMK2/ GADPH	p-MYPT1/ GADPH	Caspase-3/ GADPH
Control	/	0. 09±0.01	0. 10±0.03	0. 06±0.01	0. 08±0.01	0. 09±0.01	0. 12±0.00
Model	/	0. 44±0.04^##^	0. 50±0.01^##^	0. 55±0.02^##^	0. 56±0.04^##^	0. 56±0.01^##^	0. 52±0.04^##^
Low-dose group of Guipi Decoction	0. 5 g /mL	0. 36±0.01*	0. 39±0.01**	0. 43±0.02**	0. 49±0.03	0. 46±0.03*	0. 41±0.01*
Dose group of Guipi Decoction	1 g /mL	0. 18±0.01**	0. 34±0.01**	0. 20±0.01**	0. 26±0.04**	0. 25±0.01**	0. 22±0.01**
High-dose group of Guipi Decoction	2 g /mL	0. 24±0.01**	0. 21±0.02**	0. 30±0.01**	0. 32±0.01**	0. 39±0.02**	0. 27±0.01**
Sildenafil group	5 mg/kg	0. 17±0.01**	0. 27±0.02**	0. 36±0.01**	0. 34±0.01**	0. 19±0.02**	0. 17±0.01**

**Note:** Compared with the control, ^##^*P*<0.01; compared with the model, *P<0.05, **P<0.01.

**Table 10:** Immunohistochemical detection of protein expression of rat corpus cavernosum tissue.

Group	Dosage	RhoA	ROCK1	Caspase-3
Control	/	27. 91±7.23	36. 20±2.04	29. 41±12.64
Model	/	169. 76±6.29^##^	208. 14±12.71^##^	238. 81±35.35^##^
Low-dose group of Guipi Decoction	0. 5 g /mL	128. 27±3.84**	180. 02±14.67	190. 15±22.55
Dose group of Guipi Decoction	1 g /mL	80. 57±5.41**	85. 93±18.29**	61. 80±14.98**
High-dose group of Guipi Decoction	2 g /mL	95. 27±10.92**	134. 57±1.49**	67. 33±5.24**
Sildenafil group	5 mg/kg	82. 25±15.99**	99. 64±20.10**	94. 29±9.99**

**Note:** Compared with the control, ^##^*P*<0.01; compared with the model, **P<0.01.

## Data Availability

All the data supporting the findings of the study are provided within the article.

## LIST OF ABBREVIATIONS

ED: Erectile Dysfunction
DM: Diabetes Mellitus
DMED: Decoction ameliorates Diabetic Erectile Dysfunction
PDE5: Phosphodiesterase Type 5
TC: Total Cholesterol
BUN: Urea Nitrogen
SOD: Superoxide Dismutase
